# Association of Polymorphisms in NHEJ Pathway Genes with HIV-1 Infection and AIDS Progression in a Northern Chinese MSM Population

**DOI:** 10.1155/2022/5126867

**Published:** 2022-10-19

**Authors:** Xuelong Zhang, Xi Wang, Han Mo, Yuanting Hu, Yi Yang, Xun Yang, Jiawei Wu, Bangquan Liu, Lidan Xu, Haiming Sun, Xueyuan Jia, Ping Wang, Kaili Wang, Wenjing Sun, Songbin Fu, Yuandong Qiao

**Affiliations:** ^1^Key Laboratory of Preservation of Human Genetic Resources and Disease Control in China (Harbin Medical University), Ministry of Education, Harbin 150081, China; ^2^Laboratory of Medical Genetics, Harbin Medical University, Harbin 150081, China; ^3^Department of Gastroenterology, The First Affiliated Hospital, Harbin Medical University, Harbin 150001, China; ^4^Infectious Disease Hospital of Heilongjiang Province, Harbin 150500, China

## Abstract

**Background and Aims:**

Men who have sex with men (MSM) are at high risk of HIV infection. The nonhomologous end joining (NHEJ) pathway is the main way of double-stranded DNA break (DSB) repair in the higher eukaryotes and can repair the DSB timely at any time in cell cycle. It is also indicated that the NHEJ pathway is associated with HIV-1 infection since the DSB in host genome DNA occurs in the process of HIV-1 integration. The aim of the present investigation was to evaluate associations of single-nucleotide polymorphisms (SNPs) in NHEJ pathway genes with susceptibility to HIV-1 infection and AIDS progression among MSM residing in northern China.

**Methods:**

A total of 481 HIV-1 seropositive men and 493 HIV-1 seronegative men were included in this case-control study. Genotyping of 22 SNPs in NHEJ pathway genes was performed using the SNPscan™ Kit.

**Results:**

Positive associations were observed between *XRCC6* rs132770 and *XRCC4* rs1056503 genotypes and the susceptibility to HIV-1 infection. In gene-gene interaction analysis, significant SNP-SNP interactions of *XRCC6* and *XRCC4* genetic variations were found to play a potential role in the risk of HIV-1 infection. In stratified analysis, *XRCC5* rs16855458 was significantly associated with CD4+ T cell counts in AIDS patients, whereas *LIG4* rs1805388 was linked to the clinical phases of AIDS patients.

**Conclusions:**

NHEJ gene polymorphisms can be considered to be risk factors of HIV-1 infection and AIDS progression in the northern Chinese MSM population.

## 1. Introduction

Acquired immune deficiency syndrome (AIDS) due to the infection of human immunodeficiency virus (HIV) is a chronic infectious disease and continues to be a major global public health issue. There were approximately 37.7 million people globally and 1.045 million people in China living with HIV by the end of 2020 [1]. The significant increase in the proportion of behavior spread of men who have sex with men (MSM) is the dominant pathway of all kinds of HIV infection routes. The individuals with different susceptibility to HIV infection and clinical disease progression arise from different genetic backgrounds of the host [2]. The finding of AIDS-related genes with single-nucleotide polymorphisms (SNPs) is an important breakthrough that can help us to explore the role of host genetic background in HIV infection, reveal the pathogenesis of AIDS, predict the disease process, and develop new drugs and vaccines [3].

Double-stranded DNA break (DSB) is one of the main reasons for the gene mutation and chromosome break and plays an important role in tumorigenesis and progression of tumors [4]. The nonhomologous end joining (NHEJ) pathway is the main approach of DSB repair (DSBR) in the higher eukaryotes and can repair DSBs timely at any time in cell cycle [5, 6]. There are five core genes (*XRCC7*, *XRCC6*, *XRCC5*, *XRCC4*, and *LIG4*) in the NHEJ pathway that encodes five proteins (DNA-PK, Ku70, Ku80, XRCC4, and LIG4), respectively. Studies have shown that NHEJ gene polymorphisms are associated with susceptibility to a wide variety of cancers and disease progression. For instance, *XRCC7* gene polymorphisms play an important role in prostate cancer [7], bladder cancer [8], liver cancer [9], thyroid cancer [10], and lung cancer [11]. The other gene polymorphisms such as *XRCC4*, *XRCC5*, *XRCC6*, and *LIG4* SNPs are also associated with many different types of cancers [12–15].

It has been indicated that the NHEJ pathway is associated with HIV-1 infection because the DSB in host genome DNA occurs in the process of HIV-1 integration [16]. However, the role of SNPs in NHEJ genes and their importance in HIV-1 infection and AIDS progression remain unclear. In this study, we conducted a case-control study in the northern Han Chinese population to investigate associations of 22 SNPs in *XRCC7*, *XRCC6*, *XRCC5*, *XRCC4*, and *LIG4* genes with the risk of HIV-1 infection and the progression of AIDS. Furthermore, a gene-gene interaction analysis was conducted to explore the role of combined effects of SNPs in the risk of HIV-1 infection.

## 2. Materials and Methods

### 2.1. Subjects

A total of 481 HIV-1 seropositive men and 493 health controls were selected for this study. The study participants were all of Han descents and had lived in Harbin, Heilongjiang Province, in North China for at least three generations. All participants were not genetically related within three generations.

481 HIV-1 seropositive men were recruited from Heilongjiang Center for Disease Control and Prevention. The age of the subjects ranged from 16 to 75 years (mean age ± SD, 35.3 ± 11.55), and the average CD4+ T lymphocyte count at that time point was 335 cells/*μ*l (range, 3-1038 cells/*μ*l). All subjects had acquired HIV-1 infection through male-male homosexual transmission. These patients were categorized as category 1 (T lymphocytes < 350 cells/*μ*l) or category 2 (T lymphocytes > 350 cells/*μ*l) by the CD4+ T lymphocyte count and as category A (clinical phase III+IV) or category B (clinical phase I+II) by the clinical stage.

493 HIV-1 seronegative men age-matched to the HIV-1 patients were randomly selected as the control group from the comprehensive medical examination population of the Second Affiliated Hospital of Harbin Medical University. The age of the uninfected controls ranged from 16 to 75 years (mean age ± SD, 35.3 ± 11.59). All participants provided informed consent approved by local ethics review board.

### 2.2. SNP Selection and Genotyping

22 candidate SNPs in NHEJ pathway genes were included in the present study. Among them, two SNPs (rs7830743 and rs7003908) were from *XRCC7*, four SNPs (rs132770, rs5751129, rs2267437, and rs132774) were from *XRCC6*, eight SNPs (rs828907, rs705649, rs16855458, rs3770502, rs9288516, rs3835, rs1051677, and rs2440) were from *XRCC5*, six SNPs (rs1056503, rs6869366, rs2075685, rs10040363, rs963248, and rs35268) were from *XRCC4*, and two SNPs (rs1805388 and rs1805389) were from *LIG4*.

Genomic DNA was extracted from 200 *μ*l of peripheral blood of all participants using the QIAamp blood kit (Qiagen, Germany) according to the manufacturer's protocol. All 22 SNPs were genotyped in 481 HIV-1-infected and 493 HIV-1-uninfected individuals using a custom-designed 48-Plex SNPscan™ Kit (supplied by Genesky Bio-technologies Inc., Shanghai, China), according to the method of high-throughput SNP genotyping utilizing double ligation and multiplex fluorescence PCR. For quality control, a 5% random sample of cases and controls was genotyped twice to verify the genotyping accuracy; the reproducibility was 100%.

### 2.3. Statistical Analysis

The genotype and allele frequencies were calculated through directly counting the numbers after the genotypes of the cases and controls were determined. A chi-square test was used for examining the deviation from Hardy-Weinberg's equilibrium (HWE) for all SNPs of the control group, the association between genotype frequencies and susceptibility to HIV-1 infection, and the association between the genotype frequencies and clinical features (such as the CD4+ T lymphocyte count and clinical stage) of the case group. Odds ratios (ORs) and 95% confidence intervals (95% CI) were estimated as the relative risk associated with SNPs. The generalized multifactor dimensionality reduction (GMDR) software [17] was applied to assess SNP-SNP interactions. SPSS 23.0 software (IBM-SPSS, Inc., Chicago, IL, USA) was used for all statistical analyses. The analyses of linkage disequilibrium (LD) and the haplotype frequencies were performed using the HaploView software [18]. The differences with a *P* value less than 0.05 were considered statistically significant.

## 3. Results

### 3.1. Hardy-Weinberg Equilibrium Test

The success rates of genotyping were >98% for all SNPs. As shown in Table 1, all 22 SNPs did not deviate from the Hardy-Weinberg equilibrium in the control group (*P* > 0.05).

### 3.2. Associations of NHEJ Gene Polymorphisms with HIV-1 Infection

**Figure 1:**
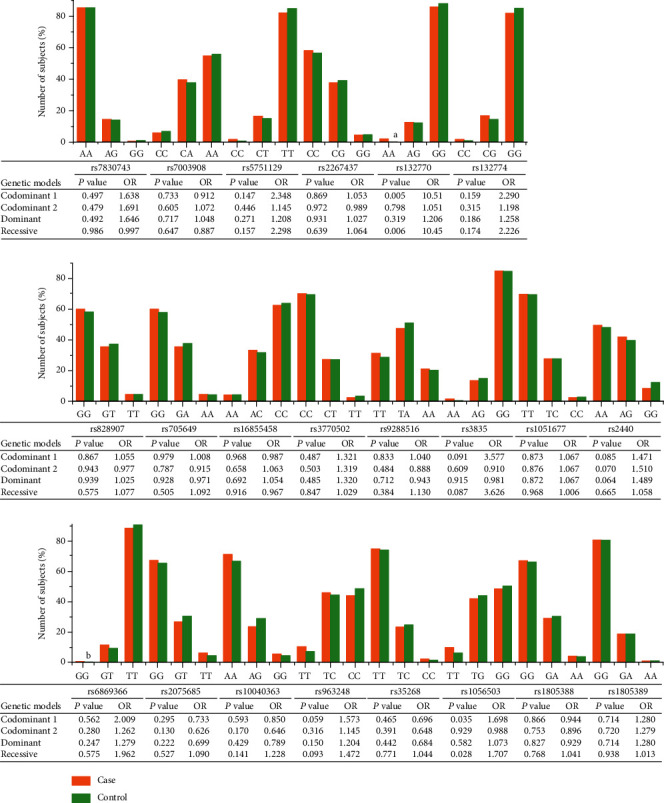
The genotype distribution map of NHEJ gene polymorphisms and association analysis of HIV-1 infection risk. The bar marked by the letters a and b corresponds to the ordinate of the minimum value of 0.2%. Codominant 1, the first column homozygote versus the third column homozygote. Codominant 2, heterozygote versus the third column homozygote. Bold italic values indicate statistical significance.

To explore the possible associations, the genotype distribution of 22 SNPs was investigated. Then, differences of genotype frequencies between cases and controls were analyzed under three genetic models (codominant model, dominant model, and recessive model). As shown in Figure 1, a significant association was found for *XRCC6* rs132770 under codominant (*P* = 0.005, OR = 10.51, 95% CI: 2.000-55.251) and recessive (*P* = 0.006, OR = 10.45, 95% CI: 1.986-54.933) genetic models. In addition, the genotype TT of *XRCC4* rs1056503 showed significant association with increased susceptibility of HIV-1 infection in the codominant model (TT vs. GG, *P* = 0.035, OR = 1.698, 95% CI: 1.037-2.779) and recessive model (TT vs. TG+GG, *P* = 0.028, OR = 1.707, 95% CI: 1.060-2.750). However, no association with HIV-1 infection was observed in any genetic model for the remaining 20 SNPs (*P* > 0.05).

### 3.3. Analysis of the SNP-SNP Interaction

**Figure 2:**
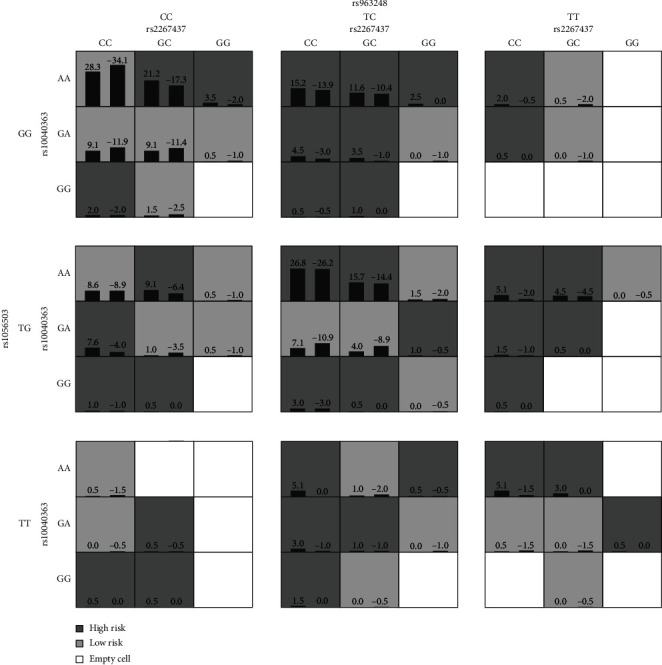
The best four-locus SNP-SNP interaction model identified by the generalized multifactor dimensionality reduction method. High-risk cells are in dark, low-risk cells are in grey, and empty cells are indicated by no shading. In each cell, the left bar represents case while the right bar represents control. The heights of the bars are proportional to the sum of samples in each group. Note that the patterns of high-risk and low-risk cells differ across each of the different multilocus dimensions, presenting evidence of SNP-SNP interaction or epistasis.

The GMDR method was used to study the association of 10 SNPs in *XRCC6* and *XRCC4* genes with high-order interactions on HIV-1 infection. Through a 10-fold cross-validation, the best four-locus model involving *XRCC6* (rs2267437) and *XRCC4* (rs10040363, rs963248, and rs1056503) was identified (Figure 2). In order to obtain the ORs for joint effects of the four SNPs on HIV-1 infection, traditional statistical methods were applied to this four-locus model to aid in interpretation, which identified three significant genotype combinations from all possible high-risk genotype combinations. In this four-locus (rs1056503-rs2267437-rs10040363-rs963248) model, the ORs for three significant high-risk genotype combinations (TT)-(CC)-(AG/GG)-(TC/CC), (TT)-(CC)-(AA)-(TC/CC), and (TT)-(CC)-(AA)-(TT) were 6.667 (*P* = 0.035), 7.333 (*P* = 0.026), and 6.667 (*P* = 0.035), respectively (Table 2).

### 3.4. Analysis of Haplotype Associations

LD between SNPs in NHEJ genes was analyzed using HaploView software. There was strong LD among four SNPs in *XRCC6* gene, eight SNPs in *XRCC5* gene, six SNPs in *XRCC4* gene, and two SNPs in *LIG4* gene, respectively. There were no significant differences in frequencies of all haplotypes between HIV-1-infected cases and healthy controls (*P* > 0.05).  Table 3 shows all blocks and haplotypes identified and the frequencies of these haplotypes.

### 3.5. Associations of NHEJ Gene SNPs with CD4+ T Cell Count and Clinical Phase in AIDS Patients

To investigate the relationship between NHEJ gene polymorphisms and AIDS progression, differences in allele frequencies were analyzed between subgroups of HIV-1-infected cases which were divided using CD4+ T lymphocyte count and clinical stage as indicators, respectively. The CD4+ T cell counts of HIV-1-infected cases ranged from 3 to 1038 cells/*μ*l (mean ± SD, 335.57 ± 198.79). The associations between SNPs and CD4+ T cell counts were used to assess the influence of gene polymorphisms on the immunity status of patients. As shown in Table 4, there were significant differences in genotype frequencies between different subgroups of cases for *XRCC5* rs16855458 and *LIG4* rs1805388 (*P* < 0.05). In detail, the subjects with AA or AC of rs16855458 have a significantly lower CD4+ T lymphocyte count, compared to subjects with CC genotype (*P* = 0.025, OR = 1.538, 95% CI: 1.054-2.243). The subjects with AA or AG of rs1805388 have a later clinical stage of AIDS, compared to subjects with GG genotype (*P* = 0.036, OR = 1.506, 95% CI: 1.027-2.209). However, other SNPs were not associated with the CD4+ T lymphocyte count and clinical stages (*P* > 0.05). These results suggested that rs16855458 and rs1805388 were associated with the clinical features and progression of AIDS in the northern Chinese population.

## 4. Discussion

According to the molecular mechanism of HIV-1 infection, viral DNA is inserted into the host genomic DNA in the process of HIV-1 integration. The integration process was equivalent to genomic DNA with DSBs in host cells under the action of HIV-1, and then, the signal of damage repair would start the NHEJ pathway. For example, the DNA-PK protein interacts with HIV-1 Tat to regulate HIV-1 replication and transcription [19, 20]. Therefore, we believed that the NHEJ genes were involved in HIV-1 infection and the disease progression. To the best of our knowledge, this comprehensive study is the first to systematically evaluate the association between the polymorphisms in NHEJ genes and the susceptibility to HIV-1 infection and the progression of AIDS.

In this study, the differences of genotype frequencies of *XRCC6* rs132770 and *XRCC4* rs1056503 were found between the cases and the controls under different genetic models. Our results implied a positive association of SNPs in NEHJ genes with the susceptibility to HIV-1 infection in the northern Chinese MSM population. The *XRCC6* gene encodes Ku70 protein, which functions as a single-stranded DNA- and ATP-dependent helicase and may be involved in the repair of nonhomologous DNA ends such as that required for DSB repair. The Ku70 protein also interacts with HIV-1 integrase and is beneficial to virus integration and replication in the process of the HIV-1 infection [21, 22]. Given that rs132770 locates close to the translation starting point in the *XRCC6* promoter, one of the possible reasons for the positive association is that rs132770 affects the expression of Ku70 mRNA; or rs132770 may be in high linkage with some functional variants conferring the etiology of HIV-1 infection. Similar to our findings, it has been reported that different *XRCC6* genotypes may contribute to susceptibility to another disease related to virus infection, namely, hepatocellular carcinoma (HCC) [23–25].

The *XRCC4* gene encodes XRCC4 protein, which can activate and enhance the activity of LIG4 protein and play an important role in NEHJ repair pathway [26]. Recently, *XRCC4* SNPs have been reported to be associated with the risk of a variety of diseases. For example, one study found that *XRCC4* mutations may lead to the occurrence of small head dwarfism [27]. Several other studies have shown that SNPs in *XRCC4* gene could affect the susceptibility and progression of virus-related HCC [28–30]. Our study implicated that *XRCC4* rs1056503 was associated with HIV-1 infection, which was consistent with the above reports. Rs1056503 is located in the 5′ regulatory region of *XRCC4* gene, which may cause changes in mRNA expression level and XRCC4 protein function. Then, functional changes in XRCC4 protein may affect NHEJ biological processes in DSBR. Further experimental assay should be performed to solidify our speculations. In addition, in the analysis of SNP-SNP interaction, our results provide evidence for a four-locus interaction between *XRCC6* and *XRCC4* variants in the risk of HIV-1 infection and further highlight the role of multilocus effects in the genetic component of HIV-1 infection.

As an indicator of AIDS clinical characteristics, CD4+ T cell count reflects the number of immune cells in patients. The AIDS patients with CD4+ T cell count less than 350 cells/*μ*l should be given antiretroviral therapy or other treatments according to the World Health Organization (WHO) [31–33]. In the present study, we found a significant difference in frequencies of *XRCC5* rs16855458 genotypes between the two subgroups of cases, where genotypes AA and AC were associated with lower numbers of CD4+ T cells. These results suggest that *XRCC5* rs16855458 is involved in the progression of AIDS. The *XRCC5* gene encodes Ku80 protein which forms a Ku heterodimer with Ku70 protein. Functional studies showed that changes in expression levels of Ku80 protein are the main reason of tumor development and can be used as a predictor of patient survival as well as treatment outcome [34, 35]. In the process of HIV-1 infection, the *XRCC5* gene is closely related to HIV-1 integration and translation [36–38]. We propose that the rs16855458 in *XRCC5* intron may regulate the transcription and expression of the *XRCC5* by alternative splicing, which interacts with HIV-1 to promote its integration and translation, leading to the decrease in the CD4+ T lymphocyte count and the AIDS acceleration. Similar to our findings, the polymorphisms of *XRCC5* gene have also been reported to be associated with virus-related HCC [24].

In this study, the HIV-1 seropositive cases were divided into two subgroups based on clinical stage, which is a clinical feature of AIDS and directly reflects the disease progression. The clinical symptoms of patients in phases I and II are mild and just show HIV-1 antibody positive. On the contrary, patients in phases III and IV have serious clinical symptoms such as nervous system lesions, continuous fever and diarrhea, sepsis, and various kinds of tumors caused by the loss of immune functions and should be timely given the antiretroviral therapy or other treatments. The results of our study revealed that there was a significant difference in genotype frequencies of *LIG4* rs1805388 between MSM cases in clinical phase I+II and those in clinical phase III+IV, and AA/AG genotypes could significantly promote the disease progression of AIDS. The *LIG4* gene encodes LIG4 protein, which connects the DSB end and completes NHEJ repair. Previous studies have shown that *LIG4* gene polymorphisms are associated with many clinical features of lung and ovarian cancer, such as treatment outcome, progression-free survival, and overall survival [39, 40]. Mutations in the *LIG4* gene can not only lead to abnormal development of immune defects but also cause severe combined immunodeficiency disease in normal individuals [41]. The rs1805388 is located in the exon region of *LIG4* gene, which is a missense mutation of threonine and isoleucine. Here, we propose that the reason for this association was the functional changes of LIG4 protein resulting from the genetic variant directly affecting the clinical stage of AIDS.

Several limitations of this study should be considered. First, there is a lack of information on critical factors in MSM cases, including history of injection drug use, clinical data on viral loads, and other clinical manifestations. Second, cases and controls were not exposed to the same conditions, because we could not collect samples of healthy MSM controls due to privacy regulations.

For future studies, we recommend that the findings of this study should be expanded to other ethnic groups in different regions in the world, beyond the northern Chinese Han population.

## 5. Conclusions

The study confirmed that NHEJ gene polymorphisms played an important role in HIV-1 infection and AIDS progression among MSM populations in northern China. Our study opens a new field for further investigation of underlying functional mechanisms of the association between NHEJ gene polymorphisms and HIV-1/AIDS.

## Figures and Tables

**Table 1:** Hardy-Weinberg equilibrium test for 22 NHEJ gene SNPs in controls.

Gene	Chr^a^	SNPs	Major/minor allele	*P* for HWET^b^
XRCC7	8	rs7830743	A/G	0.248
	8	rs7003908	A/C	0.780
XRCC6	22	rs5751129	T/C	0.677
	22	rs2267437	C/G	0.178
	22	rs132770	G/A	0.468
	22	rs132774	G/C	0.568
XRCC5	2	rs828907	G/T	0.307
	2	rs705649	G/A	0.185
	2	rs16855458	C/A	0.762
	2	rs3770502	C/T	0.501
	2	rs9288516	T/A	0.504
	2	rs3835	G/A	0.529
	2	rs1051677	T/C	0.920
	2	rs2440	A/G	0.055
XRCC4	5	rs6869366	T/G	0.936
	5	rs2075685	G/T	0.476
	5	rs10040363	A/G	0.247
	5	rs963248	C/T	0.127
	5	rs35268	T/C	0.397
	5	rs1056503	G/T	0.051
LIG4	13	rs1805388	G/A	0.810
	13	rs1805389	G/A	0.994

^a^Chr: chromosome; ^b^Hardy-Weinberg equilibrium test.

**Table 2:** Combined effects of rs1056503, rs2267437, rs10040363, and rs963248 on HIV-1 infection.

rs1056503	rs2267437	rs10040363	rs963248	*P* value	OR (95% CI)
TT	CG+GG	AG+GG	TC+CC	—	1.000
TT	CC	AG+GG	TC+CC	*0.035*	6.667 (1.145-38.833)
TT	CG+GG	AA	TC+CC	0.848	1.200 (0.185-7.770)
TT	CG+GG	AG+GG	TT	1.000	1.000 (0.125-7.995)
TT	CC	AA	TC+CC	*0.026*	7.333 (1.272-42.294)
TT	CC	AG+GG	TT	0.756	0.667 (0.051-8.639)
TT	CG+GG	AA	TT	0.642	24.556 (1.991-302.866)
TT	CC	AA	TT	*0.035*	6.667 (1.145-38.833)

Italicized values indicate statistical significance.

**Table 3:** The frequencies of haplotypes of NHEJ genes in cases and controls.

Gene	Haplotype	Frequency	Haplotype frequencies in the cases	Haplotype frequencies in the controls	Chi-square	*P*
XRCC6	TCGG	0.671	0.665	0.677	0.309	0.578
	TGGG	0.237	0.233	0.241	0.175	0.675
	CCAC	0.070	0.081	0.060	3.181	0.075
	CCGC	0.021	0.021	0.022	0.012	0.912
XRCC5	Block 1					
	GG	0.771	0.777	0.765	0.335	0.563
	TA	0.223	0.219	0.227	0.169	0.681
	Block 2					
	TG	0.468	0.470	0.466	0.040	0.841
	AG	0.452	0.448	0.456	0.149	0.699
	TA	0.080	0.082	0.078	0.115	0.735
	Block 3					
	TA	0.693	0.705	0.683	1.101	0.294
	CG	0.165	0.164	0.166	0.009	0.923
	TG	0.142	0.132	0.152	1.649	0.199
XRCC4	Block 1					
	TG	0.806	0.806	0.805	0.007	0.931
	TT	0.141	0.135	0.147	0.597	0.440
	GT	0.053	0.059	0.048	1.095	0.295
	Block 2					
	CT	0.555	0.536	0.573	2.552	0.110
	TT	0.308	0.327	0.290	3.095	0.079
	CC	0.133	0.132	0.134	0.020	0.888
LIG4	GG	0.814	0.815	0.812	0.035	0.852
	AA	0.102	0.101	0.104	0.030	0.863
	AG	0.084	0.084	0.084	0.005	0.941

**Table 4:** Associations between 22 SNPs in NHEJ genes and clinical features of AIDS.

Gene	Gene polymorphisms	Genotype	CD4+ T lymphocyte count^a^		*P*	OR (95% CI)	Clinical stage^b^		*P*	OR (95% CI)
			<350 cells/*μ*l	>350 cells/*μ*l			Phase III+IV	Phase I+II		
XRCC7	rs7830743	GG+AG	37	35	0.550	0.858 (0.519-1.418)	29	43	0.438	0.817 (0.491-1.361)
		AA	223	181			184	223		
	rs7003908	CC+CA	125	92	0.232	1.248 (0.868-1.795)	100	118	0.572	1.110 (0.773-1.594)
		AA	135	124			113	148		
XRCC6	rs5751129	CC+CT	49	39	0.825	1.054 (0.662-1.679)	40	48	0.837	1.050 (0.660-1.671)
		TT	211	177			173	218		
	rs2267437	GG+CG	118	82	0.103	1.358 (0.940-1.961)	95	107	0.335	1.196 (0.831-1.723)
		CC	142	134			118	159		
	rs132770	AA+AG	38	31	0.908	1.031 (0.617-1.722)	27	43	0.282	0.752 (0.448-1.264)
		GG	220	185			187	224		
	rs132774	CC+CG	50	39	0.743	1.081 (0.679-1.719)	40	49	0.920	1.024 (0.645-1.627)
		GG	210	177			173	217		
XRCC5	rs828907	TT+GT	104	86	0.967	1.008 (0.697-1.457)	92	99	0.185	1.283 (0.888-1.853)
		GG	156	130			121	167		
	rs705649	AA+GA	105	85	0.819	1.044 (0.722-1.509)	92	99	0.185	1.283 (0.888-1.853)
		GG	155	131			121	167		
	rs16855458	AA+AC	109	69	*0.025*	1.538 (1.054-2.243)	87	92	0.160	1.306 (0.900-1.895)
		CC	151	147			126	174		
	rs3770502	TT+CT	81	61	0.489	1.150 (0.774-1.708)	66	76	0.499	1.146 (0.772-1.700)
		CC	179	155			144	190		
	rs9288516	AA+TA	183	145	0.445	1.164 (0.789-1.717)	144	185	0.649	0.914 (0.620-1.347)
		TT	77	71			69	81		
	rs3835	AA+AG	36	35	0.495	0.839 (0.506-1.390)	33	39	0.804	1.066 (0.645-1.763)
		GG	222	181			181	228		
	rs1051677	CC+TC	83	61	0.384	1.192 (0.803-1.768)	68	77	0.481	1.151 (0.778-1.703)
		TT	177	155			145	189		
	rs2440	GG+AG	127	113	0.451	0.870 (0.607-1.249)	96	136	0.767	0.947 (0.660-1.358)
		AA	133	103			107	130		
XRCC4	rs6869366	GG+GT	36	20	0.124	1.575 (0.883-2.811)	23	33	0.587	0.855 (0.485-1.505)
		TT	224	196			190	233		
	rs2075685	TT+GT	77	80	0.087	0.715 (0.487-1.050)	62	95	0.126	0.739 (0.502-1.089)
		GG	183	136			151	171		
	rs10040363	GG+AG	72	66	0.510	0.875 (0.588-1.302)	59	79	0.613	0.902 (0.605-1.345)
		AA	187	150			154	186		
	rs963248	TT+TC	136	132	0.054	0.698 (0.484-1.007)	114	155	0.298	0.825 (0.574-1.186)
		CC	124	84			99	111		
	rs35268	CC+TC	70	51	0.409	1.192 (0.786-1.808)	60	61	0.191	1.318 (0.872-1.992)
		TT	190	165			153	205		
	rs1056503	TT+TG	128	118	0.241	0.805 (0.561-1.156)	107	140	0.602	0.909 (0.633-1.303)
		GG	132	98			106	126		
LIG4	rs1805388	AA+AG	87	69	0.726	1.071 (0.729-1.574)	81	77	*0.036*	1.506 (1.027-2.209)
		GG	173	147			132	189		
	rs1805389	AA+AG	52	39	0.591	1.135 (0.716-1.799)	48	45	0.124	1.429 (0.907-2.249)
		GG	208	177			165	221		

Italicized values indicate statistical significance. ^a^The CD4+ T lymphocyte counts were divided into two groups: category 1, <350 cells/*μ*l, and category 2, >350 cells/*μ*l. ^b^Clinical stage: category A, clinical phase III+IV, and category B, clinical phase I+II.

## Data Availability

The data used to support the findings of this study are available from the corresponding author upon request.

## References

[1] Wang T., Gu Y., Ran L., Tan X., Peng S. (2022). Ways of HIV transmission in China: the effect of age, period, and cohort. *Frontiers in Public Health*.

[2] McLaren P. J., Carrington M. (2015). The impact of host genetic variation on infection with HIV-1. *Nature Immunology*.

[3] van Manen D., van 't Wout A. B., Schuitemaker H. (2012). Genome-wide association studies on HIV susceptibility, pathogenesis and pharmacogenomics. *Retrovirology*.

[4] Sishc B. J., Davis A. J. (2017). The role of the core non-homologous end joining factors in carcinogenesis and cancer. *Cancers (Basel).*.

[5] Chang H. H. Y., Pannunzio N. R., Adachi N., Lieber M. R. (2017). Non-homologous DNA end joining and alternative pathways to double-strand break repair. *Nature Reviews. Molecular Cell Biology*.

[6] Zhao X., Wei C., Li J. (2017). Cell cycle-dependent control of homologous recombination. *Acta Biochim Biophys Sin (Shanghai).*.

[7] Xiao M., Shen Y., Chen L., Liao Z., Wen F. (2014). The rs 7003908 (T>G) polymorphism in the XRCC7 gene and the risk of cancers. *Molecular Biology Reports*.

[8] Zhi Y., Yu J., Liu Y. (2012). Interaction between polymorphisms of DNA repair genes significantly modulated bladder cancer risk. *International Journal of Medical Sciences*.

[9] Aboul Enein A. A., Khaled I. A. A., Khorshied M. M. (2020). Genetic variations in DNA-repair genes (XRCC1, 3, and 7) and the susceptibility to hepatocellular carcinoma in a cohort of Egyptians. *Journal of Medical Virology*.

[10] Jamshidi M., Farnoosh G., Mohammadi P. S., Rafiee F., Boroujeni A. S., Mahmoudian-Sani M. R. (2021). Genetic variants and risk of thyroid cancer among Iranian patients. *Horm Mol Biol Clin Investig.*.

[11] Singh A., Singh N., Behera D., Sharma S. (2018). Role of polymorphic XRCC6 (Ku70)/XRCC7 (DNA-PKcs) genes towards susceptibility and prognosis of lung cancer patients undergoing platinum based doublet chemotherapy. *Molecular Biology Reports*.

[12] Garcia J. A., Kalacas N. A., Sy O. T., Ramos M. C., Albano P. M. (2019). XRCC4 c.1394G>T single nucleotide polymorphisms and breast cancer risk among Filipinos. *Asian Pacific Journal of Cancer Prevention*.

[13] He X., Zhu X., Li L. (2016). The relationship between polymorphisms of XRCC5 genes with astrocytoma prognosis in the Han Chinese population. *Oncotarget*.

[14] Mumbrekar K. D., Goutham H. V., Vadhiraja B. M., Bola Sadashiva S. R. (2016). Polymorphisms in double strand break repair related genes influence radiosensitivity phenotype in lymphocytes from healthy individuals. *DNA Repair (Amst)*.

[15] Yin M., Liao Z., Liu Z. (2012). Genetic variants of the nonhomologous end joining gene LIG4 and severe radiation pneumonitis in nonsmall cell lung cancer patients treated with definitive radiotherapy. *Cancer*.

[16] Knyazhanskaya E., Anisenko A., Shadrina O. (2019). NHEJ pathway is involved in post-integrational DNA repair due to Ku70 binding to HIV-1 integrase. *Retrovirology*.

[17] Xu H. M., Xu L. F., Hou T. T. (2016). GMDR: versatile software for detecting gene-gene and gene-environ- ment interactions underlying complex traits. *Current Genomics*.

[18] Barrett J. C., Fry B., Maller J., Daly M. J. (2005). Haploview: analysis and visualization of LD and haplotype maps. *Bioinformatics*.

[19] Tyagi S., Ochem A., Tyagi M. (2011). DNA-dependent protein kinase interacts functionally with the RNA polymerase II complex recruited at the human immunodeficiency virus (HIV) long terminal repeat and plays an important role in HIV gene expression. *The Journal of General Virology*.

[20] Zhang S. M., Zhang H., Yang T. Y. (2014). Interaction between HIV-1 Tat and DNA-PKcs modulates HIV transcription and class switch recombination. *International Journal of Biological Sciences*.

[21] Ilgova E., Galkin S., Khrenova M., Serebryakova M., Gottikh M., Anisenko A. (2022). Complex of HIV-1 integrase with cellular Ku protein: interaction interface and search for inhibitors. *International Journal of Molecular Sciences*.

[22] Warrilow D., Tachedjian G., Harrich D. (2009). Maturation of the HIV reverse transcription complex: putting the jigsaw together. *Reviews in Medical Virology*.

[23] Hsu C. M., Yang M. D., Chang W. S. (2013). The contribution of XRCC6/Ku70 to hepatocellular carcinoma in Taiwan. *Anticancer Research*.

[24] Li R., Yang Y., An Y. (2011). Genetic polymorphisms in DNA double-strand break repair genes XRCC5, XRCC6 and susceptibility to hepatocellular carcinoma. *Carcinogenesis*.

[25] Jia J., Ren J., Yan D., Xiao L., Sun R. (2015). Association between the XRCC6 polymorphisms and cancer risks: a systematic review and meta-analysis. *Medicine (Baltimore)*.

[26] Tang J., Li Z., Wu Q., Irfan M., Li W., Liu X. (2022). Role of paralogue of XRCC4 and XLF in DNA damage repair and cancer development. *Frontiers in Immunology*.

[27] Murray J. E., van der Burg M., IJspeert H. (2015). Mutations in the NHEJ component XRCC4 cause primordial dwarfism. *American Journal of Human Genetics*.

[28] Jung S. W., Park N. H., Shin J. W. (2012). Polymorphisms of DNA repair genes in Korean hepatocellular carcinoma patients with chronic hepatitis B: possible implications on survival. *Journal of Hepatology*.

[29] Long X. D., Zhao D., Wang C. (2013). Genetic polymorphisms in DNA repair genes XRCC4 and XRCC5 and aflatoxin B1-related hepatocellular carcinoma. *Epidemiology*.

[30] Lu J., Wang X. Z., Zhang T. Q. (2017). Prognostic significance of XRCC4 expression in hepatocellular carcinoma. *Oncotarget*.

[31] Palella F. J., Deloria-Knoll M., Chmiel J. S. (2003). Survival benefit of initiating antiretroviral therapy in HIV-infected persons in different CD4+ cell strata. *Annals of Internal Medicine*.

[32] Siegfried N., Uthman O. A., Rutherford G. W., Cochrane HIV/AIDS Group (2010). Optimal time for initiation of antiretroviral therapy in asymptomatic, HIV-infected, treatment-naive adults. *Cochrane Database of Systematic Reviews*.

[33] When To Start Consortium, Sterne J. A., May M. (2009). Timing of initiation of antiretroviral therapy in AIDS-free HIV-1-infected patients: a collaborative analysis of 18 HIV cohort studies. *Lancet*.

[34] Li W., Xie C., Yang Z., Chen J., Lu N. H. (2013). Abnormal DNA-PKcs and Ku 70/80 expression may promote malignant pathological processes in gastric carcinoma. *World Journal of Gastroenterology*.

[35] Tseng S. H., Yang C. C., Yu E. H. (2015). K14-EGFP-miR-31 transgenic mice have high susceptibility to chemical-induced squamous cell tumorigenesis that is associating with Ku80 repression. *International Journal of Cancer*.

[36] Jean M. J., Power D., Kong W., Huang H., Santoso N., Zhu J. (2017). Identification of HIV-1 Tat-associated proteins contributing to HIV-1 transcription and latency. *Viruses*.

[37] Jeanson L., Subra F., Vaganay S. (2002). Effect of Ku80 depletion on the preintegrative steps of HIV-1 replication in human cells. *Virology*.

[38] Manic G., Maurin-Marlin A., Laurent F. (2013). Impact of the Ku complex on HIV-1 expression and latency. *PLoS One*.

[39] Xu F., Han J. C., Zhang Y. J. (2015). Associations of LIG4 and HSPB1 genetic polymorphisms with risk of radiation-induced lung injury in lung cancer patients treated with radiotherapy. *BioMed Research International*.

[40] Ali R., Alabdullah M., Algethami M. (2021). Ligase 1 is a predictor of platinum resistance and its blockade is synthetically lethal in XRCC1 deficient epithelial ovarian cancers. *Theranostics.*.

[41] Luo X., Liu Q., Jiang J. (2021). Characterization of a cohort of patients with LIG4 deficiency reveals the founder effect of p.R278L, unique to the Chinese population. *Frontiers in Immunology*.

[42] Zhang X. L., Wang K., Mo H. (2021). Associations of the polymorphisms of the NHEJ pathway genes with HIV-1 infection and aids progression among men who have sex with men in northern China. *Research Square.*.

